# Efficacy and safety of vonoprazan-based bismuth quadruple therapy for first-line *Helicobacter pylori* eradication: A large-scale, real-world study

**DOI:** 10.1097/MD.0000000000040170

**Published:** 2024-10-18

**Authors:** Jihai Zhou, Li Jia, Zhu Liu, Wenen Zhao, Lifeng Liu, Xin Chen, Fengyu Gao

**Affiliations:** a Department of Gastroenterology, Shandong Provincial Maternal and Child Health Care Hospital Affiliated to Qingdao University, Jinan, China; b Department of Gastroenterology and Hepatology, Tianjin Medical University General Hospital, Tianjin Institute of Digestive Disease, Tianjin Key Laboratory of Digestive Diseases, Tianjin, China.

**Keywords:** adverse event, eradication rate, *Helicobacter pylori*, real-world, vonoprazan

## Abstract

Vonoprazan (VPZ) has been shown to have superior acid-inhibitory effects compared to proton pump inhibitors (PPIs). However, there is a paucity of research examining the efficacy of vonoprazan-based bismuth quadruple therapy (VBQT) in the eradication of primary *Helicobacter pylori* infection. This study aimed to evaluate the effectiveness and safety of VBQT as a first-line treatment for *H pylori* eradication. This retrospective, real-world, single-arm study included consecutive treatment-naive patients who received VBQT (VPZ 20 mg, amoxicillin 1000 mg, clarithromycin 500 mg, bismuth potassium citrate 220 mg, all administered twice daily for 14 days) for *H pylori* eradication between March 1, 2021, and May 30, 2023. The study included both outpatients and inpatients. Eradication rates were assessed using ^13^C-urea breath tests or ^14^C-urea breath tests performed 4 to 6 weeks after treatment. The primary outcomes included eradication rates, adverse events, and treatment compliance. A total of 612 *H pylori*-infected patients were included in the study. The intention-to-treat (ITT), modified ITT (MITT), and per-protocol analyses showed *H pylori* eradication rates of 84.3% (95% CI: 812% to 87.1%), 95.9% (95% CI: 93.9% to 97.4%), and 96.4% (95% CI: 94.4% to 97.8%), respectively. In the ITT analysis, the adverse event rate was 12.7%, and the treatment compliance rate was 96.9%. In real-world practice, the VBQT regimen demonstrates excellent efficacy and favorable tolerability as a first-line therapy for *H pylori* eradication.

## 1. Introduction

*Helicobacter pylori* is a pathogenic bacterium known for its role in causing inflammation of the gastric mucosa and various gastric-related diseases, including chronic gastritis, gastric adenocarcinoma, and gastric mucosa-associated lymphoid tissue lymphoma (MALT).^[1]^ With a global infection rate exceeding 50%,^[2]^ the eradication of *H pylori* has become crucial in preventing the progression of chronic gastritis, preventing peptic ulcer recurrence and reducing the risk of gastric cancer.^[3]^ International guidelines now recommend eradication therapy for all individuals infected with *H pylori*.^[4,5]^ Consequently, achieving effective eradication of *H pylori* is a critical challenge faced by clinicians.

The current Chinese management guideline for *H pylori* strongly endorses the use of a 14-day bismuth-containing quadruple therapy (BQT) based on a proton pump inhibitor (PPI) as the first-line treatment.^[4–6]^ However, the recommended BQT has shown eradication rates ranging from 79.5% to 86.6%,^[7]^ falling below the internationally agreed-upon standard of 90%. This highlights the insufficiency of current treatment approaches to meet the clinical needs in China.

The decreasing eradication rate of PPI-based regimens is primarily attributed to increasing antibiotic resistance and inadequate suppression of gastric acid production.^[8,9]^ Proton pump inhibitors (PPIs), influenced by dietary factors and CYP2C19 gene polymorphisms, exhibit a delayed onset (requiring 75–100 hours to achieve maximum acid inhibition) and a short half-life. Therefore, there is an urgent need to use excellent acid suppressants in *H pylori* eradication strategies.

One promising acid suppressant is vonoprazan (VPZ), a novel potassium-competitive acid blocker (P-CAB) that competitively inhibits the binding of potassium ions and H^+^/K^+^-ATPase in gastric parietal cells. Unlike PPIs, VPZ does not require acid activation and is not influenced by CYP2C19 genetic variations. It exerts stronger and more sustained inhibitory effects on gastric acid production.

Several studies have demonstrated that VPZ-based triple therapy has higher *H pylori* eradication rates than PPI-based triple therapy.^[10,11]^ However, Only 2 clinical trials have compared the efficacy of BQT based on VPZ and PPI in *H pylori* eradication. One study involving Asian patients with duodenal ulcers, including those from China, demonstrated that VPZ-based BQT (VBQT) achieved a significantly higher eradication rate than PPI-based BQT (PBQT) (91.5% vs 86.8%, *P* = .047).^[12]^ Another study showed the non-inferiority of VBQT compared to PBQT (94.1% vs 91.1%, *P* < .001).^[13]^ In our previous study,^[14]^ we compared the efficacy of the VBQT with VPZ-based dual therapy in treatment-naive patients with *H pylori* infection. The results indicated that the eradication rate of VPZ-based dual therapy was non-inferior to that of VBQT (96.8% vs 94.4%, *P* < .025). However, due to the use of propensity score matching analysis, only 78 patients per group were included in the final analysis. Moreover, the latest Chinese guidelines for *H pylori* treatment recommend VBQT as first-line and rescue regimens for *H pylori* infection. Nevertheless, the quality of evidence for this recommendation is low, rendering it a weak recommendation. The small sample sizes, homogeneous patient populations, and discordant conclusions underscore the need for larger real-world studies to confirm the efficacy and safety of 14-day VBQT regimens.

Real-world studies complement randomized controlled trials by providing evidence from clinical practice. With this in mind, we conducted the present study to evaluate the efficacy and safety of VPZ-containing BQT regimens as first-line therapy for *H pylori* eradication.

## 2. Materials and methods

### 2.1. Study design and ethics

This study was designed as a retrospective, single-arm study conducted at the Shandong Provincial Maternal and Child Health Hospital affiliated to Qingdao University between March 1, 2021, and May 30, 2023. The study adhered to the ethical guidelines of the Declaration of Helsinki and was approved by the Ethics Committee of Qingdao University Affiliated Hospital of Shandong Province Maternal and Child Healthcare Hospital (Ethics Committee Approval No: 2023-043). Informed consent was not required from patients as this was a non-interventional study based on real-world data.

### 2.2. Study population and treatment regimens

Consecutive patients diagnosed with *H pylori* infection via at least 1 of the following methods: ^13^C-urea breath test (^13^C-UBT), ^14^C-urea breath test (^14^C-UBT), *H pylori* antibody detection (no history of *H pylori* eradication.), or pathological examination, and treated with VBQT were eligible for inclusion. The VBQT regimen consisted of VPZ 20 mg, amoxicillin 1000 mg, clarithromycin 500 mg, bismuth potassium citrate 220 mg, VPZ and bismuth citrate were taken 30 minutes before meals, while amoxicillin and clarithromycin were taken immediately after meals, all twice daily for 14 days. Patients were excluded if they met any of the following criteria: a history of prior *H pylori* eradication treatment; non to study medications; loss to follow-up or refusal of follow-up; recent use of PPI, P-CAB, bismuth, or antibiotic therapy within the past 4 weeks prior to taking the antimicrobial drugs; a history of gastric resection; pregnancy, breastfeeding, or a known allergy to the study drugs.

### 2.3. Study procedures

The primary endpoint of this study was the *H pylori* eradication rate. The secondary endpoints included the frequency and severity of adverse events. The ^13^C-UBT or ^14^C-UBT was performed 4 to 6 weeks after treatment to confirm eradication success if the result was negative.

The study population comprised both outpatients and inpatients. Demographic data were collected, including patient type, contact number, name, age at diagnosis, gender, height, weight, body mass index (BMI), smoking history, alcohol drinking, history, number of comorbidities (hypertension, coronary heart disease, diabetes, cerebrovascular disease, thyroid disease, etc), endoscopic findings, compliance, adverse events and severity through medical record review and necessary telephone interviews.

Adverse events such as diarrhea, nausea, vomiting, constipation, abdominal bloating, abdominal pain, abdominal discomfort, skin rash, pruritus, bitter taste, fatigue, anorexia, and giddy were documented. The severity of adverse events was categorized as mild, moderate, or severe. Mild referred to adverse events not requiring treatment; moderate referred to adverse events requiring treatment for control but not hospitalization; severe referred to patients experiencing adverse symptoms that required hospitalization or resulted in study-related death. Poor compliance was defined as taking <80% of the prescribed medication.

Analyses were performed using intention-to-treat (ITT), modified intention-to-treat (MITT), and per-protocol (PP) sets. ITT analysis included patients who took the treatment medication at least once, while patients who did not undergo follow-up ^13^C-UBT or ^14^C-UBT were considered treatment failures. MITT analysis included patients who took the treatment medication at least once and underwent follow-up ^13^C-UBT or ^14^C-UBT. PP analysis included patients who took more than 80% of the prescribed medication and underwent follow-up ^13^C-UBT or ^14^C-UBT.

### 2.4. Sample size calculation and statistical analysis

As this study was an observational cohort study, formal sample size, and power calculations were not conducted. Categorical variables were presented as frequencies and percentages, while continuous variables were presented as means and standard deviations. Multivariable analysis using the enter method was conducted to assess factors associated with successful *H pylori* eradication, with a significance level of *P* < .10 in the univariate analysis. All analyses were performed using the Statistical Package for the Social Sciences (SPSS) version 26.0 software package.

## 3. Results

### 3.1. Patient enrollment and baseline characteristics

**Figure 1.**
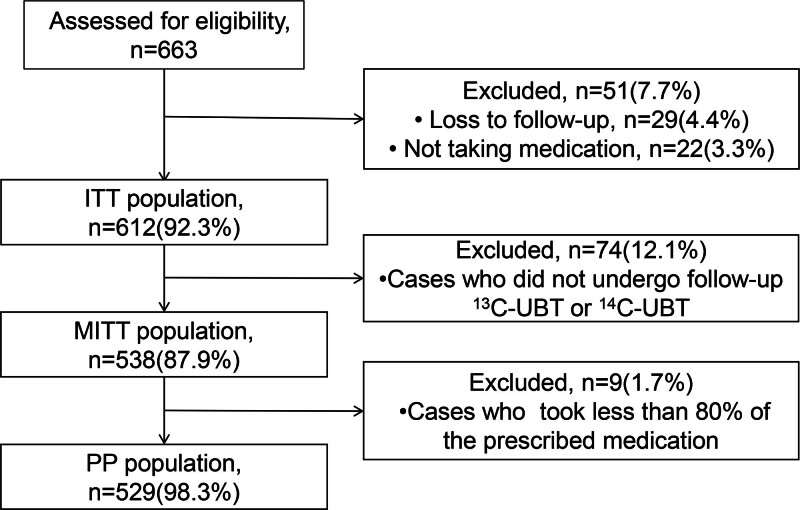
Flow for the selection of patients. ITT, intention-to-treat; MITT, modified ITT analysis, PP, per protocol.

The flowchart of the study protocol is shown in Figure 1. A total of 663 patients met the inclusion criteria in this study, of which 51 cases (7.7%) refused treatment or were lost to follow-up and were excluded from the study. A total of 612 patients were included in the ITT analysis, comprising 265 males (43.3%) and 347 females (56.7%), with a mean age of 45.33 years. Among the participants, there were 182 (29.7%) inpatients and 430 (70.3%) outpatients, with 392 cases (64.1%) undergoing gastroscopy examination. Seventy-four (12.1%) patients did not undergo follow-up ^13^C-UBT or ^14^C-UBT; therefore, 538 patients were included in the modified MITT analysis. Nine (1.7%) patients had medication compliance below 80%. Consequently, 529 patients were included in the PP analysis. Baseline characteristics of the study participants are presented in Table 1.

**Table 1:** Patient demographics and characteristics.

Characteristics	VBQT (N = 612) n (%)
Patient type	
Outpatient	430 (70.3%)
Inpatient	182 (29.7%)
Age (yr, mean ± SD)	45.33 ± 13.19
Gender	
Male	265 (43.3%)
Female	347 (56.7%)
BMI (kg/m^2^, mean ± SD)	24.24 ± 3.14
Smoking	97 (15.8%)
Alcohol drinking	180 (29.4%)
Number of comorbidities	
0	494 (81.1%)
1	82 (13.4%)
2	25 (4.1%)
More than 2	8 (1.4%)
Endoscopic findings	
No gastroscopy	220 (35.9%)
Only non-atrophic gastritis	167 (27.3%)
Only atrophic gastritis	108 (17.6%)
Mainly gastritis and esophagitis	24 (4.0%)
Mainly gastritis and gastric ulcer and/or duodenal ulcer	45 (7.4%)
Mainly gastritis and globoduodenitis	20 (3.3%)
Mainly gastritis and cardiac polyp and/or gastric polyp	25 (4.2%)
Other diagnosis	2 (0.3%)
Compliance	593 (96.9%)

BMI = body mass index, VBQT = vonoprazan-based bismuth-containing quadruple therapy.

### 3.2. Eradication rates

A total of 516 patients successfully eradicated *H pylori*. The eradication rates in the ITT, MITT, and PP analyses were 84.3% (95% CI: 81.2 to 87.1%, 516/612), 95.9% (95% CI: 93.9% to 97.4%, 516/538), 96.4% (95% CI: 94,4% to 97.8%, 510/529), respectively, as shown in Table 2.

**Table 2:** *Helicobacter pylori* eradication rates.

Analysis	VBQT
PP	96.4% (510/529)
95% CI	94.4% to 97.8%
MITT	95.9% (516/538)
95% CI	93.9% to 97.4%
ITT	84.3% (516/612)
95% CI	81.2% to 87.1%

CI = confidence interval, ITT = intention-to-treat, MITT = modified ITT analysis, PP = per protocol, VBQT = vonoprazan-based bismuth-containing quadruple therapy.

### 3.3. Adverse events and patient compliance

In the ITT analysis, 78 cases (12.7%) experienced adverse events, mainly including nausea (19, 3.1%), skin rash (10, 1.6%), diarrhea (14, 2.3%), abdominal discomfort (7, 1.1%), abdominal pain (3, 0.5%), bloating (9, 1.5%), vomiting (2, 0.3%), fatigue (3, 0.5%). The majority of adverse events were mild (81.3%), as shown in Table 3. Three cases discontinued the medication due to moderate skin or vomiting, and 2 cases were hospitalized for severe diarrhea or skin rash. The medication compliance rate was 96.9% (593/612).

**Table 3:** The adverse events of VBQT.

	VBQT (N = 612) n (%)
No. of patients with adverse events	78 (12.7%)
Diarrhoea	14 (2.3%)
Nausea	19 (3.1%)
Vomiting	2 (0.3%)
Fatigue	3 (0.5%)
Abdominal discomfort	7 (1.1%)
Abdominal pain	3 (0.5%)
Abdominal bloating	9 (1.5%)
Skin rash	10 (1.6%)
Bitter mouth	4 (0.7%)
giddy	2 (0.3%)
Constipation	3 (0.5%)
Pruritus	1 (0.2%)
Anorexia	1 (0.2%)
hiccup	1 (0.2%)
bad breath	1 (0.2%)
Acid regurgitation	1 (0.2%)
Total no. of adverse events	80
Mild	65
Moderate	13
Severe	2

VBQT = Vonoprazan-based bismuth-containing quadruple therapy.

### 3.4. Factor analysis

Univariate analysis was initially conducted, including factors such as patient gender, age, BMI, patient type, smoking history, alcohol consumption history, adverse events, endoscopy findings, comorbidities, and patient compliance to investigate potential factors affecting *H pylori* eradication efficacy. Finally, with the eradication rate of *H pylori* as the dependent variable, logistic regression analysis was performed, and the multivariable regression analysis showed that poor compliance was a risk factor for successful *H pylori* eradication (Table 4).

**Table 4:** Factors affecting *Helicobacter pylori* eradication efficacy.

Factors	Univariate analysis		Multivariate analysis	
	OR (95% CI)	*P* value	OR (95% CI)	*P* value
Patient type Inpatient versus outpatient	0.656 (0.262–1.647)	.370		
Age, yr		.440		
40 to 60 versus 18 to 40	1.483 (0.580–3.789)	.411		
>60 versus 18 to 40	2.403 (0.532–10.865)	.255		
Gender Female versus male	3.173 (1.272–7.916)	.013	1.410 (0.427–4.658)	.573
BMI, kg/m^2^ >25versus ≤ 25	0.458 (0.194-1.080)	.075	0.663 (0.266–1.655)	.379
Smoking Yes versus no	0.742 (0.244-2.253)	.598		
Alcohol drinking Yes versus no	0.250 (0.105-0.599)	.002	0.327 (0.105–1.016)	.053
Number of comorbidities	1.302 (0.377–4.496)	.676		
Endoscopic findings		.716		
Only non-atrophic gastritis versus no gastroscopy	0.595 (0.211–1.679)	.327		
Only atrophic gastritis versus no gastroscopy	0.735 (0.210–2.577)	.631		
Complex diagnosis versus no gastroscopy	1.120 (0.283–4.426)	.872		
Compliance Good versus poor	13.42 (3.118–57.768)	.000	12.671 (2.602–61.703)	.002
Adverse events Yes versus no	0.881 (0.253–3.062)	.842		
Severity of adverse events		.907		
No versus moderate	0.000	.999		
Mild versus moderate	0.000	.999		

BMI = body mass index, CI = confidence interval, OR = odds ratio.

## 4. Discussions

To our knowledge, this is the first real-world study to explore the efficacy and safety of 14-day VBQT regimens as first-line treatment for *H pylori* eradication. Our findings demonstrate a remarkable *H pylori* eradication rate of 96.4% based on PP analysis, surpassing the recommended threshold of 90% outlined in the *H pylori* eradication guidelines.

Previous 2 studies conducted in China^[12,13]^ have also explored the efficacy of a 14-day VPZ-containing BQT regimen, reporting eradication rates ranging from 91.5% to 94.1%, which were either superior or non-inferior to a 14-day PPI-based BQT regimen (ranging from 86.8% to 91.1%). However, both studies had smaller sample sizes, and 1 of them included only duodenal ulcer patients,^[12]^ while the other study was a retrospective controlled study that might have introduced substantial selection bias.^[13]^ As a result, there is a need for large real-world evidence to validate and complement these findings. Moreover, our study demonstrated a higher eradication rate than these 2 previous studies, which may be attributed to the larger sample size and the inclusion of patients with various gastrointestinal diseases, not limited to just duodenal ulcers. Although this study shares some data with our previous research,^[14]^ the earlier study primarily focused on comparing VPZ-based dual therapy and VBQT in treatment-naive *H pylori*-infected patients. In contrast, the current study provides a more extensive real-world validation of the efficacy and safety of VBQT as a first-line treatment for *H pylori* eradication, utilizing a larger sample size.

The excellent eradication rate achieved with the 14-day VPZ-containing BQT regimen can be attributed to several factors. Firstly, VPZ effectively maintains gastric pH above 5 for 99% of the time within 24 hours,^[10,15,16]^ increasing the concentration and stability of amoxicillin and clarithromycin in the gastric mucosa,^[17,18]^ and lowering the minimum inhibitory concentration. Secondly, *H pylori* enters a replicative state at pH values between 6 and 8, rendering it more susceptible to growth-dependent antibiotics.^[19]^ Thirdly, VPZ achieves maximum acid suppression more rapidly and maintains it for a longer duration compared to PPIs. Unlike PPIs that take 3 to 5 days to achieve maximum and stable gastric acid inhibition, VPZ achieves it within 4 hours. Consequently, the VPZ-based regimen ensures optimal pH levels for *H pylori* treatment throughout the entire course of therapy.^[20,21]^ Lastly, unlike PPIs, the pharmacokinetic characteristics of VPZ are not influenced by CYP2C19 polymorphisms, providing consistent drug exposure and efficacy across different patient populations.

To improve eradication rates, replacing clarithromycin with alternative antibiotics with lower-resistance in VPZ-containing regimens may be beneficial. A study from Japan demonstrated that VAC regimens with metronidazole instead of clarithromycin achieved a high eradication rate of 98.5%.^[22]^ In China, primary resistance rates of *H pylori* to clarithromycin, levofloxacin, amoxicillin, furazolidone, tetracycline, and metronidazole are 37.00%, 34.21%, 2.20%, 1.61%, 1.18%, and 87.87%, respectively.^[23]^ Replacing clarithromycin with lower-resistance antibiotics like furazolidone or tetracycline in VPZ-based BQT regimens may further improve eradication rates for both first-line and rescue treatments. However, multi-center randomized controlled trials are necessary to validate these findings.

Theoretically, it is feasible to shorten the duration of VBQT regimens while maintaining effective eradication rates. Shorter treatment durations offer advantages such as minimizing disruption to gut microflora, improving patient compliance, reducing costs, and decreasing antibiotic usage. Several studies in Japan have shown that, despite being 50% shorter in duration, the 7-day VAC regimen achieves eradication rates ranging from 87.4% to 96.7%, which is comparable to the 14-day PPI triple or BQT regimens.^[24,25]^ The faster acid inhibition of VPZ may allow for a shorter duration of *H pylori* treatment compared to PPIs. A recent study in China demonstrated that a 10-day VPZ-based BQT regimen (with VPZ 20mg daily) achieved comparable eradication rates to the standard 14-day regimens (98.6% vs 97.4%).^[26]^. This raises the question of whether the standard 14-day C-BQT regimen (with VPZ 20 mg twice daily) could potentially be shortened to 7 or 10 days while maintaining similar efficacy. Further investigation is needed to explore this possibility.

VBQT therapy was well tolerated and safe. The most commonly reported adverse events were nausea, diarrhea, skin rash, bloating, and abdominal discomfort. No life-threatening adverse events were reported. Previous studies indicate that the incidence of adverse events associated with VBQT is comparable to PBQT,^[13,27]^ however, the incidence of adverse events in our study was lower than in previous studies,^[13,26,28]^ potentially due to patient recall bias. Since most adverse events of the VBQT regimen were mild, patients may have underreported mild them when surveyed.

Poor compliance was the main factors for treatment failure. A meta-analysis showed communication strategies improved compliance and eradication rates.^[29]^ In our study, after receiving verbal and written medical guidance, 96.9% (593/612) of patients demonstrated good compliance.

Our study has several strengths: it included a large sample size, providing reliable statistical power; it is the first real-world study on VBQT for first-line *H pylori* eradication, reflecting actual clinical practice; multivariate regression analysis identified factors associated with eradication failure.

However, there are also some limitations: the study design was retrospective and single-arm, conducted at a single center, limiting the generalizability of the results. Prospective cohort studies involving multiple centers are needed; antibiotic susceptibility testing was not performed, precluding a comparison of eradication rates between patients with clarithromycin-sensitive and -resistant strains. Nonetheless, excellent eradication rates were achieved, and routine use of susceptibility testing is invasive and costly; a significant proportion of patients did not undergo ^13^C-UBT or ^14^C-UBT retesting. However, the study employed ITT, MITT, and PP analysis to account for this; and the current evidence for VBQT primarily comes from Asian populations. Further studies in other ethnic groups, such as populations from Western nations, are necessary to validate our findings.

## 5. Conclusion

As the first large-scale, real-world study, our research demonstrated excellent eradication rates, patient compliance, and low incidence of adverse events for VPZ-containing BQT as a first-line treatment for *H pylori* infection in the Chinese population. Further validation is needed in other ethnic groups.

## Acknowledgments

I would like to express my gratitude to the Paperpal system for their assistance in correcting the grammar in the article. I also want to thank Professor Fengyu Gao for the support with data statistics.

## Author contributions

**Conceptualization:** Jihai Zhou, Zhu Liu.

**Data curation:** Li Jia, Wenen Zhao.

**Formal analysis:** Lifeng Liu, Xin Chen.

**Methodology:** Li Jia, Zhu Liu.

**Project administration:** Fengyu Gao.

**Validation:** Xin Chen, Fengyu Gao.

**Writing – original draft:** Zhu Liu.

**Writing – review & editing:** Fengyu Gao.
